# Denoising autoencoder framework for reconstructing missing periodontal clinical records

**DOI:** 10.3389/fdmed.2026.1710316

**Published:** 2026-03-16

**Authors:** Asok Mathew, Pradeep Kumar Yadalam

**Affiliations:** 1 Department of Clinical Sciences, College of Dentistry, Centre for Medical and Bioallied Health Sciences Research, Ajman University, Ajman, United Arab Emirates; 2 Department of Periodontics, Saveetha Dental College and Hospitals, Saveetha Institute of Medical and Technical Sciences (SIMATS), Saveetha University (SIMATS), Chennai, India

**Keywords:** artificial intelligence, clinical data, missing values, periodontitis, synthetic data

## Abstract

**Introduction:**

Missing clinical data pose a significant challenge for retrospective analyses and predictive modelling in periodontal research. Traditional imputation methods often overlook the complex correlations among variables and produce implausible values. This study examines the application of generative models to reconstruct missing periodontal clinical records and outlines a comprehensive workflow that spans from data generation to evaluation.

**Methods:**

A synthetic periodontal dataset of 200 virtual patients was constructed, capturing realistic distributions of demographic factors (age, gender, smoking, and diabetes) and tooth-level measurements (probing depth, attachment loss, furcation involvement, and bleeding on probing) across eight sites. Missingness was introduced at random to 15% of the clinical variables. A denoising autoencoder with a single hidden layer of 18 neurons was trained to reconstruct the original data from corrupted inputs over 100 epochs. The model learned latent representations of the data and was then used to impute missing entries.

**Results:**

Performance was assessed by comparing reconstructed values to the original data using mean absolute error (MAE) and root mean squared error (RMSE) across individual variables and categories. Overall, MAE and RMSE were 0.61 and 0.74, respectively, with attachment loss and bleeding on probing exhibiting lower errors than probing depth and furcation involvement.

**Conclusion:**

Distribution comparisons showed that imputed values closely matched original distributions. The approach offers a practical framework for handling missing periodontal data and highlights the potential of generative models to improve data completeness without introducing unrealistic values. Limitations include the simplicity of the network architecture. Future work should explore advanced models, integrate multimodal data, and evaluate on real datasets. The workflow—from synthetic data creation and masking to training, imputation, and evaluation—serves as a template for researchers tackling missing clinical data.

## Introduction

Periodontal disease affects the tissues that surround and support the teeth, including the gums, periodontal ligament, and alveolar bone. It is one of the most prevalent chronic diseases globally and can lead to tooth loss if left untreated. Periodontal health is assessed through a combination of clinical measurements, including pocket probing depth, clinical attachment level, furcation involvement, and bleeding on probing, as well as patient factors such as age, smoking status, and systemic health conditions (1, 2). These measurements are recorded during routine dental visits and research studies to monitor disease progression and evaluate treatment effectiveness. Comprehensive clinical datasets are critical for understanding disease patterns, conducting epidemiological studies, training diagnostic algorithms, and guiding precision therapies. However, many periodontal datasets suffer from missing data, where some variables are not recorded for certain patients or tooth sites. Missingness (3) may arise from incomplete charting, variations in clinical practice patterns, data-entry errors, or record loss. Incomplete datasets hinder large-scale analyses, reduce statistical power, introduce bias, and limit the applicability of machine learning models trained on such data (4).

Missing clinical data is not a unique problem in periodontal research. Incomplete electronic health records, inconsistent data collection, and heterogeneous data sources are among the problems researchers encounter across the health care field. Conventional imputation methods, such as mean or regression imputation, can fill in missing data but often overlook the complex multivariate dependencies in biomedical data. In periodontal datasets, variables such as probing depth and attachment loss are correlated and influenced by demographics and systemic conditions, with nonlinear relationships across tooth sites. Using simple averages can distort relationships and lead to false conclusions. Recent studies explore advanced imputation methods, such as machine learning techniques, that capture interactions and non-linearities. However, imputation remains a deterministic prediction, not a generative reconstruction. Traditional methods, such as mean substitution and regression imputation, fill in missing values but ignore complex multivariate relationships. In periodontal data, variables such as probing depth and attachment loss are correlated, influenced by demographics and systemic conditions, and show non-linear patterns across tooth sites. Simply replacing missing values with averages may distort these relationships and cause inaccurate conclusions. A growing body of work has explored advanced techniques for imputing missing data, including machine learning models that capture interactions and non-linearities. Many conventional machine learning imputation methods are single-imputation, producing one dataset with deterministic predictions. Conversely, multiple imputation methods like MICE use stochastic draws for multiple datasets, enabling uncertainty propagation and pooled inference under Rubin's rules. Recent techniques, such as MICE-based ensembles (e.g., MiceForest) and uncertainty-aware models, incorporate stochastic sampling. The main difference is between deterministic single-imputation and probabilistic multiple imputation that explicitly models uncertainty data (5).

Periodontal clinical data encompass a variety of measurements collected during dental examinations. Pocket probing depth is the distance from the gingival margin to the bottom of the periodontal pocket and indicates the severity of periodontal disease. Clinical attachment level measures the position of the periodontal attachment relative to a fixed landmark on the tooth, combining probing depth and recession. Furcation involvement describes the loss of bone and soft tissue between the roots of multi-rooted teeth, categorised into grades based on the extent of attachment loss. Bleeding on probing is a binary indicator signifying bleeding observed when the periodontal probe is inserted into a periodontal pocket, reflecting inflammation. These measurements are typically recorded for each tooth or specific tooth sites (e.g., mesiobuccal, distobuccal, mid-buccal), resulting in high-dimensional clinical datasets. Additional variables such as patient age, gender, smoking status, and the presence of systemic conditions like diabetes provide context for interpreting periodontal findings. Missing outcome data in clinical trials can lead to attrition bias, compromising the validity of the results. Intention-to-treat (ITT) analysis, supported by appropriate imputation methods, is recommended over per-protocol (PP) analysis to preserve randomization and ensure credible findings (6).

A previous study demonstrated that ccGAN significantly improves EHR data imputation by modeling nonlinear, multivariate dependencies and conditioning on observed, fully annotated features. It outperforms state-of-the-art methods by up to 19.79% in imputation accuracy and 1.61% under high missingness (6, 7). Another study demonstrated that, in a COVID-19 mortality case study using EHR data with high missingness, the best results were achieved with a pipeline that combines translation and encoding imputation to capture informative missingness, along with tree-based classifiers such as random forests and gradient-boosting. This approach consistently achieved superior AUC performance, making it an ideal method for predicting outcomes in sparse clinical datasets (8). A recent study evaluated three imputation methods—GAIN (Generative Adversarial Imputation Networks), missForest, and MICE (Multiple Imputation by Chained Equations)—on two clinical datasets with 20% and 50% missing data. GAIN and missForest outperformed MICE in accuracy, with GAIN excelling at 50% missingness, especially for skewed continuous and imbalanced categorical variables. GAIN was also significantly faster than missForest with large sample sizes. Results suggest that GAIN is a more accurate, efficient, and robust method for analyzing large clinical datasets, making it a promising approach for future big data research. Previous EHR studies (ccGAN, translation–encoding pipelines, GAIN vs. missForest vs. MICE) have advanced imputation but largely overlook the unique structure of periodontal datasets with mixed variable types (7). This highlights the need for domain-specific, clinically plausible methods to reconstruct missing periodontal data. Recent generative models like autoencoders, VAEs, GANs, and diffusion models have transformed data synthesis, image generation, and representation learning by learning latent distributions, generating new samples, and reconstructing inputs. This study aims to use autoencoders to reconstruct periodontal clinical data. It explores the application of generative models to recover missing records and presents a comprehensive workflow that covers data generation through evaluation.

## Methods

### Synthetic dataset construction

To develop and evaluate our generative imputation model, we constructed a synthetic dataset of periodontal clinical data. Using synthetic data avoids privacy concerns associated with real patient records and allows us to control the distribution and missingness patterns for robust evaluation. The dataset simulates the clinical measurements collected during routine periodontal examinations, as well as demographic variables commonly used in periodontology research.

**Figure 1:**
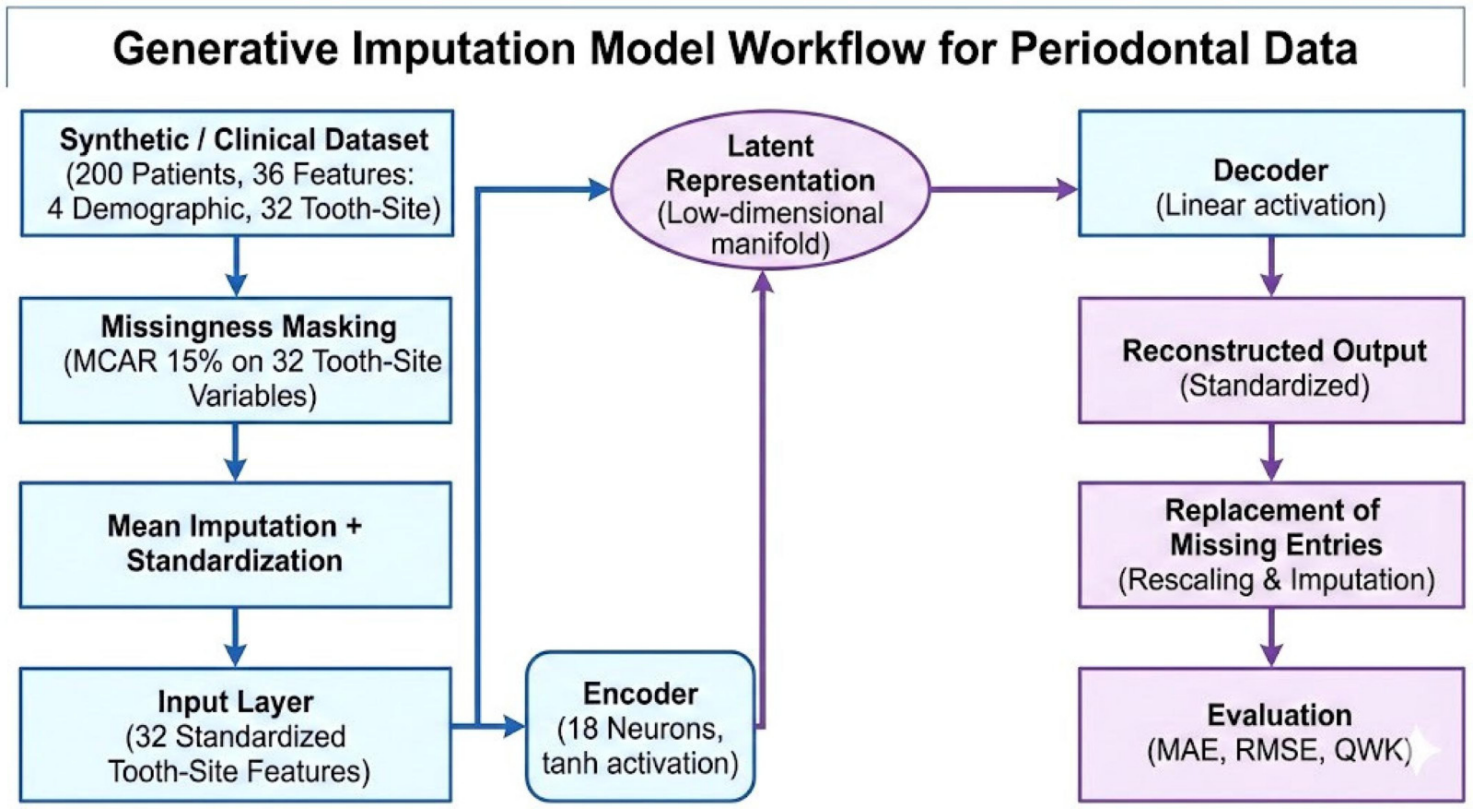
The study workflow.

We generated a cohort of two hundred virtual patients. Each patient record includes the following demographic attributes: age (an integer uniformly sampled between 18 and 80 years), gender (binary indicator coded as 0 for male and 1 for female), smoking status (binary indicator denoting current smoking, coded as 0 for non-smoker and 1 for smoker), and diabetes status (binary indicator coded as 0 for absence of diabetes and 1 for presence of diabetes). These demographic variables reflect major risk factors for periodontal disease and are essential covariates in clinical analyses (Figure 1).

For periodontal measurements, we modelled eight representative tooth sites: the upper right first incisor (UR1), upper right first molar (UR6), upper left first incisor (UL1), upper left first molar (UL6), lower right first incisor (LR1), lower right first molar (LR6), lower left first incisor (LL1), and lower left first molar (LL6). For each site, we generated four clinical variables:

**Probing Depth:** a continuous variable representing the depth of the periodontal pocket in millimetres. We simulated probing depth values from a normal distribution with a mean of 3.5 mm and a standard deviation of 1.0 mm. Values were constrained to be at least 1 mm, reflecting the minimum biologically plausible pocket depth.
**Attachment Loss:** a continuous variable representing the clinical attachment level, derived from the difference between probing depth and gingival margin location. We simulated attachment loss from a normal distribution with a mean of 2.0 mm and a standard deviation of 1.0 mm and then clipped negative values to zero. To introduce correlation between probing depth and attachment loss, we applied a linear combination: attachment loss += 0.5 × (probing depth—attachment loss). This formulation ensures that greater probing depths correspond to higher attachment loss, reflecting clinical reality.
**Furcation Involvement:** an ordinal variable indicating the degree of furcation involvement for multi-rooted teeth. We sampled furcation grades from a categorical distribution with probabilities of 60% for no furcation involvement (grade 0), 25% for slight involvement (grade I), 10% for moderate involvement (grade II), and 5% for advanced involvement (grade III).
**Bleeding on Probing:** a binary variable coded as 0 or 1, representing whether bleeding was observed when a periodontal probe was inserted into the pocket. We sampled bleeding on probing from a Bernoulli distribution with a 60% probability.

This design yields 32 tooth-site variables (eight sites × four measurements) in addition to the four demographic variables. The resulting dataset contains 36 features per patient. The synthetic values capture plausible ranges and distributions observed in clinical practice. However, they remain synthetic and are used solely for methodological demonstration.

### Inducing missingness

To evaluate the model's ability to reconstruct missing clinical data, we introduced missing values into the synthetic dataset. A missing rate of 15% was applied randomly across the 32 tooth-site variables. Specifically, for each patient and each tooth-site variable, a Bernoulli trial with probability 0.15 was used to determine whether that value was masked. Missingness was assumed to be independent of the data values, consistent with a missing completely at random (MCAR) mechanism. Although real-world missingness may depend on unobserved or observed variables (missing at random or not at random), the MCAR assumption simplifies the demonstration. It isolates the generative model's reconstruction performance.

The demographic variables (age, gender, smoking status, and diabetes) were retained for all patients because these variables are typically recorded in most clinical settings. By focusing on tooth-site variables, we created a scenario in which the model must learn the relationships among periodontal measurements and their associations with patient characteristics to impute missing entries. The resulting dataset, which included missing values, was saved for further analysis and model evaluation.

The synthetic dataset provided a controlled environment for testing reconstruction under known distributions and MCAR missingness. Since the data processing was predefined and simple, benchmarking against traditional methods was reserved for real clinical data, where performance matters more.

### Generative model architecture and training

#### Denoising autoencoder design

We implemented a denoising autoencoder using a fully connected neural network. The network takes as input a vector of 32 tooth-site variables (after standardisation) and outputs a reconstruction of these 32 variables. The architecture comprises a single hidden layer with 18 neurons that captures the latent structure of the data. The encoder transforms the input into a latent representation using a hyperbolic tangent (tanh) activation, while the decoder reconstructs the output using a linear activation. Weight matrices and biases are initialised randomly and updated during training using gradient descent.

Mathematically, let $x\in R^{32}$ Denote the input vector of scaled tooth-site measurements. The encoder computes a hidden representation *h* As:

$$h=\tanh(W_{1}x+b_{1}),$$

where $W_{1}\in R^{32\times 18}$ and $b_{1}\in R^{18}$ These are the encoder weights and biases. The decoder reconstructs the output. $\hat{x}$ As:

$$\hat{x}=W_{2}h+b_{2},$$

where $W_{2}\in R^{18\times 32}$ and $b_{2}\in R^{32}$ These are the decoder weights and biases. The reconstruction loss is measured using mean squared error:

$$L=\frac{1}{n}\overset{n}{\underset{i=1}{\sum }}∥{\hat{x}}^{\left(i\right)}-x^{\left(i\right)}{∥}^{2},$$

where *n* Is the number of training examples. During training, the input vectors are corrupted by replacing missing values with the column means (to allow the model to handle missing entries) and standardising the features. The model learns to map the noisy inputs to the original uncorrupted targets. Although more sophisticated architectures could incorporate deeper layers, variational sampling, or attention mechanisms, our design demonstrates that even a relatively simple network can learn meaningful latent representations for imputation.

The network architecture (single hidden layer with 18 neurons) was intentionally selected as a minimal proof-of-concept configuration to evaluate the feasibility of generative reconstruction under controlled conditions. Extensive hyperparameter tuning (e.g., depth variation, dropout regularization, learning rate scheduling, or alternative latent dimensionalities) was not performed, as the objective of this study was methodological demonstration rather than architectural optimization.

#### Training procedure

The training dataset included all 200 patient records with missing values filled using column means for input scaling. Standardisation involved subtracting the mean and dividing by the standard deviation, computed from the filled dataset to prevent data leakage. The training ran for 100 epochs, with each epoch including a forward pass to compute reconstruction and a backward pass to update weights via gradient descent. The learning rate was 0.01. The mean squared reconstruction loss was recorded at each epoch to monitor training.

#### Imputation and post-processing

After training, the denoising autoencoder imputed missing values in the synthetic dataset. For each patient record with missing data, we replaced missing entries with column means, standardized the data using the same method as in training, and then processed the vector through the trained encoder and decoder to reconstruct the values. These were then rescaled to original units. Missing entries in the original dataset were replaced with reconstructed values. Using the same mean and standard deviation from training ensured no bias was introduced.

MICE was implemented in Python 3.10 using statsmodels (v0.14.0) and miceforest (v5.7.0), generating five stochastic imputations with ten cycles. the predictor matrix included demographic and tooth-level variables. Continuous (probing depth, clinical attachment level) were imputed with predictive mean matching; binary (bleeding on probing) with logistic regression; and ordinal (furcation involvement) with proportional odds logistic regression. Imputed values were constrained within plausible ranges (e.g., probing depth ≥1 mm; furcation grades 0–3). KNN imputation used scikit-learn (v1.3.0) with *k* = 5 and Euclidean distance on standardized data, employing neighbor averaging for continuous and majority voting for categorical variables. All analyses used fixed random seeds for reproducibility.

#### Validation using real patient data

We developed a generative autoencoder–diffusion framework to reconstruct missing periodontal records from a synthetic cohort of 200 virtual patients. To validate this approach, we applied the same method to a real dataset of 200 periodontal patients, similar to previous studies, which included multiple tooth-level variables with approximately 12% missing values.

The real clinical dataset consisted of 200 periodontal patients retrospectively extracted from institutional electronic health records at Saveetha Dental College. The dataset consisted of fully anonymized retrospective clinical records with no identifiable patient information. As the study involved secondary analysis of de-identified data without patient contact or intervention, formal ethical approval was not required under institutional guidelines. Inclusion criteria were adults (≥18 years) with periodontitis per current classification, and complete baseline periodontal data (probing depth, CAL, mobility, bleeding). Exclusion criteria were incomplete data, recent periodontal surgery (<6 months), systemic conditions affecting periodontal health (except controlled diabetes), or records missing >50% tooth-level info. The dataset had about 12% missing data in tooth variables. Missingness was likely due to inconsistent clinical documentation and site-specific recording, rather than being completely random.

Continuous measures included bleeding on probing, probing depth, mobility, and CAL. We first split the data into an 80/20 training/test set, then randomly masked 30% of the originally observed values in the test set to create ground-truth pairs for evaluation. Four imputation methods were compared: simple mean imputation, k-nearest neighbours (KNN) imputation, multiple imputation by chained equations (MICE), and a denoising autoencoder (DAE).

### Evaluation metrics

To assess imputation quality, we compared reconstructed data to the original synthetic dataset before missingness was introduced. For each tooth-site variable and patient, we identified missing entries and calculated the absolute and squared differences between imputed and true values. The main metrics were: 1. Mean Absolute Error (MAE): the average absolute difference, indicating error magnitude. 2. Root Mean Squared Error (RMSE): the square root of the average squared difference, penalizing larger errors. We computed the MAE and RMSE for each variable and across the entire dataset, weighting errors by the number of missing entries. Variables were grouped by clinical category (probing depth, attachment loss, furcation, bleeding), and category-level MAE and RMSE were calculated to identify which measurements are harder to impute. For MICE-based imputation, MAE and RMSE were calculated within each of the five datasets by comparing imputed to ground-truth masked values. The final MAE and RMSE are averages across imputations, maintaining variability and ensuring comparability with pooled inference. MICE was implemented in Python 3.10 using the statsmodels (v0.14.0) and miceforest (v5.7.0) libraries.

## Results

The study showed that a denoising autoencoder trained for 100 epochs on a synthetic periodontal dataset effectively reconstructed missing clinical records. Its reconstruction loss decreased and plateaued, indicating successful learning. When imputing missing values, the distributions of variables like probing depths matched those in the original data, suggesting plausible imputed values. Quantitative evaluation yielded a mean absolute error of 0.61 and a root mean squared error of 0.74, with lower errors for binary variables such as bleeding and higher errors for ordinal variables such as furcation involvement. These results demonstrate that a simple model can accurately and plausibly impute missing periodontal data, highlighting variables needing more advanced models.

### Training behaviour

The denoising autoencoder was trained over 100 epochs, with the mean squared reconstruction loss steadily decreasing and plateauing. Early epochs showed rapid learning of principal components, while later stages refined subtle relationships. The low final loss indicates the network captured meaningful data structures. The training loss curve illustrates the effectiveness of gradient descent in optimizing the autoencoder for reconstruction. The loss measures reconstruction error on the scaled dataset, not direct imputation accuracy on missing data. Hence, a low training loss doesn't guarantee perfect imputation but lays a foundation for successful reconstruction.

### Distribution comparison

To assess the plausibility of the imputed values, we examined the distribution of a representative variable, probing depth at UR1. We compared the original non-missing values with imputed values for masked entries. The original histogram was bell-shaped around 3.5 mm, reflecting the mean. Imputed values had a similar, slightly narrower distribution, indicating the model captured the central tendency and variance. The overlap suggests the autoencoder produced plausible, clinically expected values. For variables such as attachment loss, furcation involvement, and bleeding, the imputed distributions mirrored the originals. Binary variables were imputed as probabilities and thresholded. Ordinal variables, such as furcation involvement, were challenging because the network output continuous values interpreted as discrete categories, but the values clustered around plausible levels, showing the model captured the distribution.

The histogram comparison in Figure 3 evaluates marginal distribution similarity under an MCAR masking mechanism, which was introduced at random. Similarity between observed and imputed marginal distributions is expected and does not indicate accurate recovery of the data's joint structure. Instead, it serves as a plausibility check, while reconstruction quality was more rigorously assessed through paired comparisons at masked locations using MAE and RMSE.

### Quantitative imputation performance

#### Overall metrics

Aggregating across all imputed entries, the overall mean absolute error (MAE) was approximately 0.61, and the overall root mean squared error (RMSE) was approximately 0.74. These values reflect the average magnitude of error across all tooth-site variables and patients. The fact that RMSE exceeds MAE indicates the presence of some larger errors, as RMSE penalises larger deviations more strongly than MAE. Nonetheless, both metrics are reasonably low given the synthetic dataset's range of values (probing depths and attachment losses typically span 0–6 mm, and furcation grades range from 0 to 3).

#### Category-Level metrics

Error metrics differed across the four clinical categories. Table 1 summarises the weighted MAE and RMSE for each category, where weights correspond to the number of missing entries for variables in that category. Categories with higher mean values (e.g., furcation involvement) naturally yielded higher absolute errors because mispredictions at a single category level translate into larger numerical differences. The lowest errors were observed for bleeding on probing, a binary variable; the model correctly imputed the presence or absence of bleeding in most cases, resulting in a low average error. Attachment loss also exhibited relatively low error compared to probing depth and furcation involvement.

**Table 1:** Category-level imputation errors.

Category	MAE	RMSE
Probing depth	0.6,971	0.8931
Attachment loss	0.5005	0.6200
Furcation	0.7926	0.9710
Bleeding on probing	0.4646	0.4943

Examining individual features revealed variability in error rates. The five features with the highest mean absolute errors were UL6_Furcation (MAE ≈ 0.95), LR1_Furcation (MAE ≈ 0.87), LL6_Furcation (MAE ≈ 0.85), LR6_Furcation (MAE ≈ .84), and LR6_ProbingDepth (MAE ≈ 0.81). These variables represented furcation involvement grades and probing depth measurements at molar sites, which likely exhibited greater variability and were more difficult to reconstruct accurately. Furcation grades are discrete and ordinal, making it difficult for a continuous-valued autoencoder to replicate the exact class boundaries precisely. Additionally, the molar sites may have wider ranges of probing depths due to anatomical differences and the higher prevalence of advanced disease in posterior teeth. Variables such as bleeding on probing and attachment loss at anterior sites showed fewer errors. The binary nature of bleeding on probing helped the model estimate probabilities accurately, and attachment loss was strongly correlated with probing depths, making it easier to reconstruct the data. This indicates that generative models perform better on continuous and binary variables than on ordinal variables. Using ordinal regression or softmax could improve results.

We generated bar charts to visualise the MAE and RMSE across individual variables and clinical categories. The MAE bar chart displays features sorted by descending MAE, highlighting the variables with the greatest imputation challenges. The category bar charts revealed that furcation involvement had the highest average error, followed by probing depth, attachment loss, and bleeding on probing. These visualisations provide an intuitive overview of where the generative model performs well and where it struggles.

#### Validation results

Model-based imputers outperformed the autoencoder on clinical data. For continuous variables, MICE had the lowest errors (MAE ≈ 1.44; RMSE ≈ 2.18), followed by KNN (MAE ≈ 1.47; RMSE ≈ 2.18). Mean imputation showed higher errors (MAE ≈ 5.10; RMSE ≈ 5.58), while DAE scored intermediate (MAE ≈ 3.41; RMSE ≈ 3.94). MICE's iterative modeling excels on real data. Among features, bleeding on probing was hardest to reconstruct (KNN MAE ≈ 8.38), while maintenance and scaling had low errors.

In the real clinical validation dataset, traditional imputation methods—especially MICE—outperformed the denoising autoencoder in reconstructing both continuous and ordinal variables. This emphasizes a key difference between synthetic and real data. The synthetic dataset, with controlled distributions and MCAR missingness, enabled the autoencoder to learn coherent representations. Conversely, real datasets exhibit structured heterogeneity, potential MAR/MNAR mechanisms, mixed variable types, and limited sample sizes, conditions in which regression-based models like MICE may better capture dependencies. Thus, while generative models offer theoretical advantages for modeling joint distributions, conventional statistical methods may be more effective for moderate missingness in structured clinical data.

These results don't invalidate the generative approach, but they suggest improvements to the model architecture and loss function. The current autoencoder uses continuous reconstruction loss for mixed variables, without feature-specific handling for ordinal or binary outcomes. Using hybrid loss functions, multi-head outputs, conditional covariate embeddings, or diffusion-based uncertainty modelling may improve performance. Larger, diverse training cohorts could also enhance latent learning. These findings suggest generative reconstruction is feasible, but conventional imputation may be more effective for real-world periodontal datasets with modest missingness and mixed variables.

The ordinal clinical state variable, imputed with the continuous variables, showed similar trends. MICE closely matched true categories (QWK ≈ 0.97; MAE ≈ 0.27), effectively capturing disease severity. KNN also performed well (QWK ≈ 0.81), while DAE often misclassified severe vs. hopeless states (QWK ≈ 0.39), and mean imputation missed variations. Overall, this validation demonstrates that while generative models can reconstruct missing periodontal data, conventional methods such as MICE may be more effective on real clinical datasets with modest missingness and mixed variables. The performance gap between synthetic and real data underscores the need to enhance the autoencoder architecture, such as incorporating more complex latent structures, feature-specific loss functions, or conditional information, to maximize the benefits of generative imputation in periodontal research.

## Discussion

**Figure 2:**
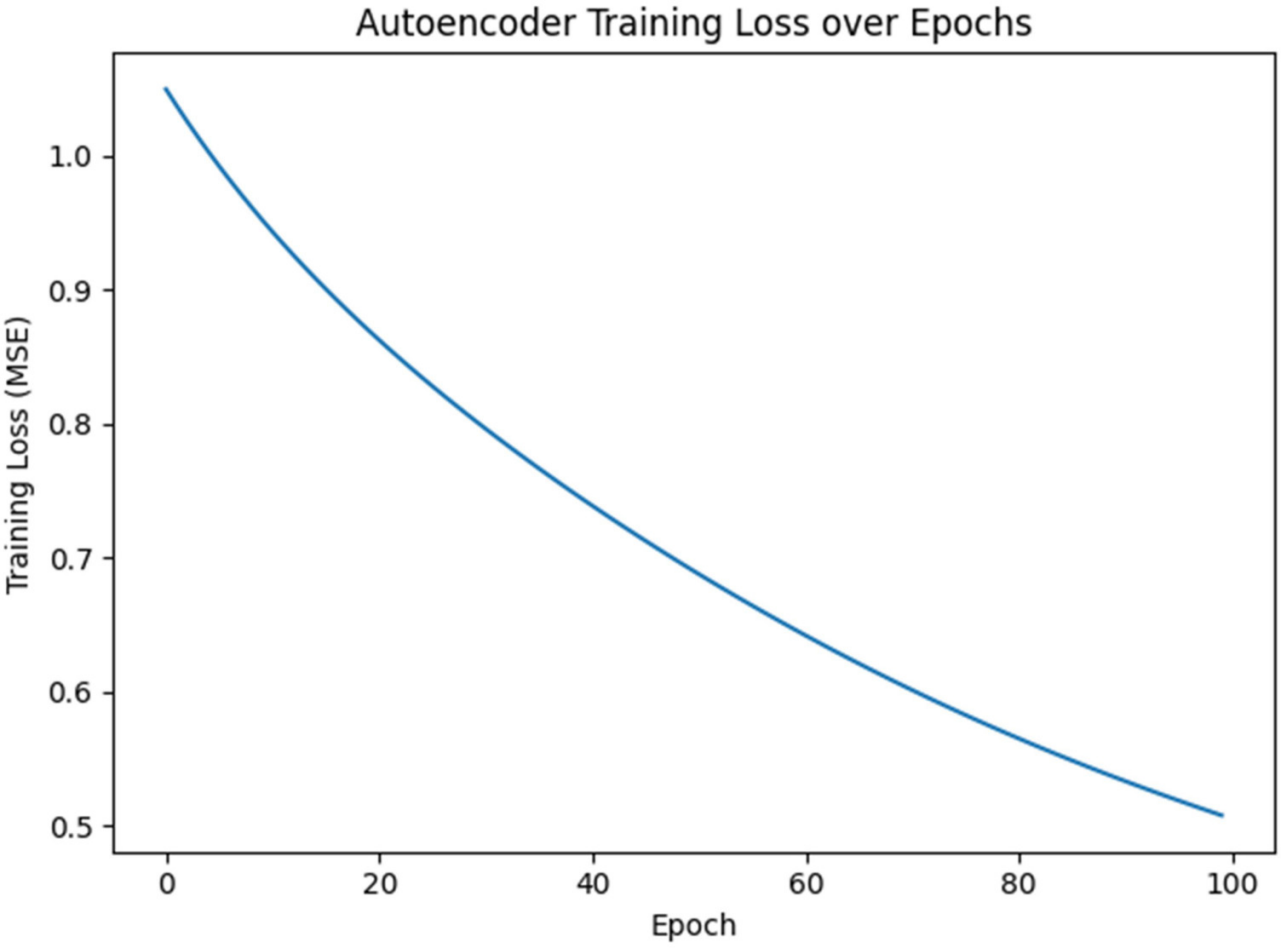
The epoch loss of the model.

**Figure 3:**
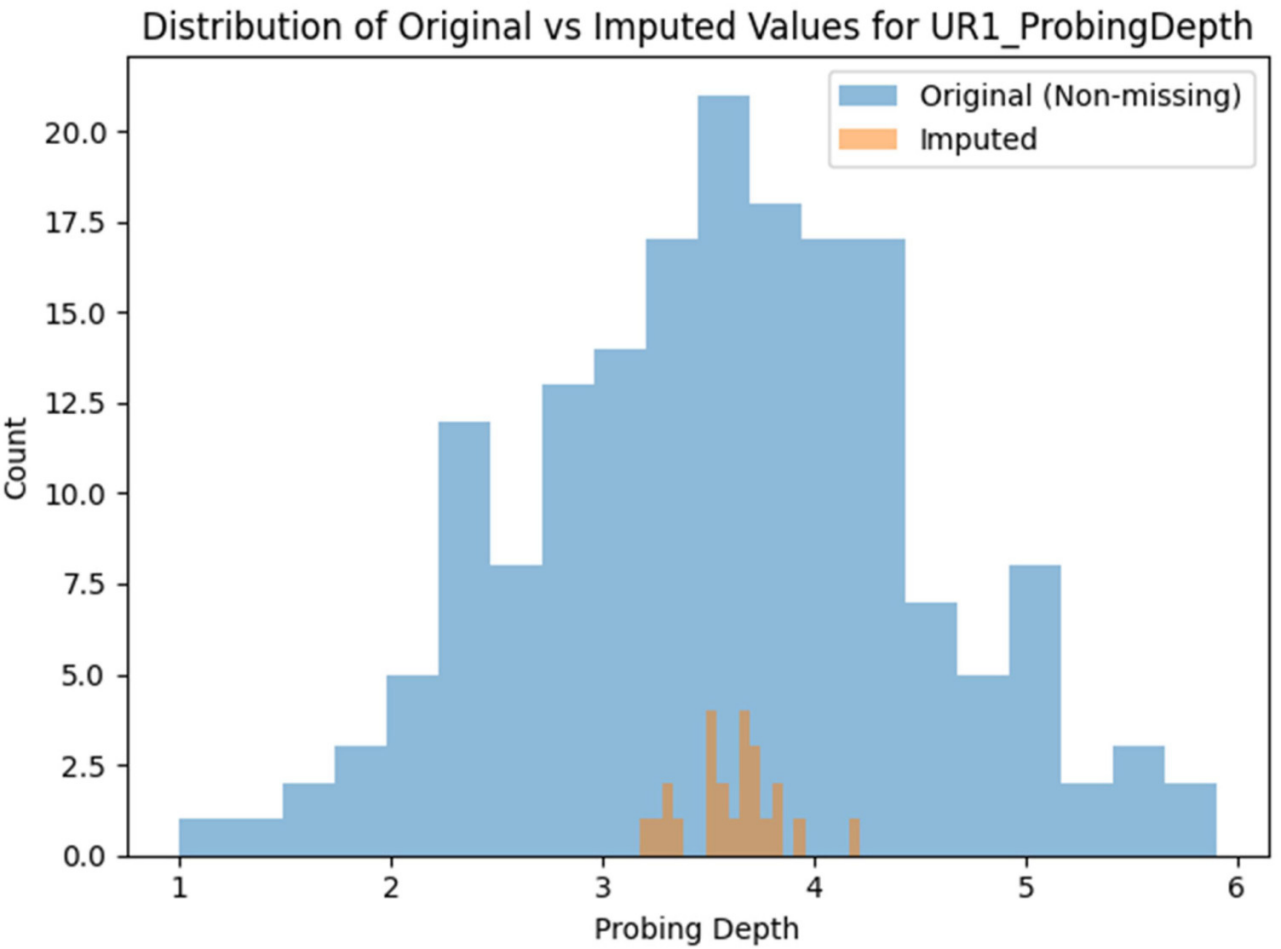
The distribution of original and imputed values.

**Figure 4:**
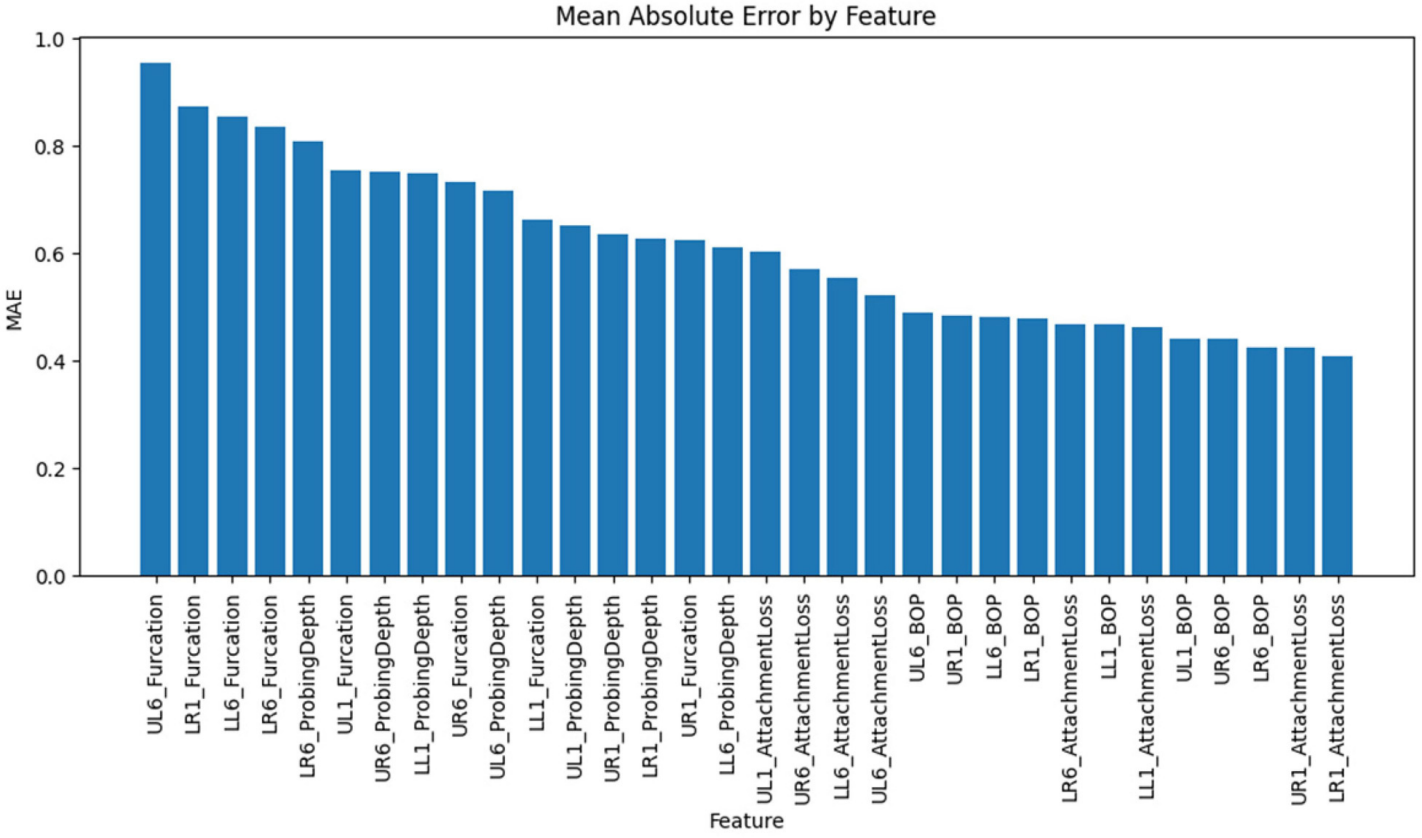
Shows the mean absolute error by feature.

**Figure 5:**
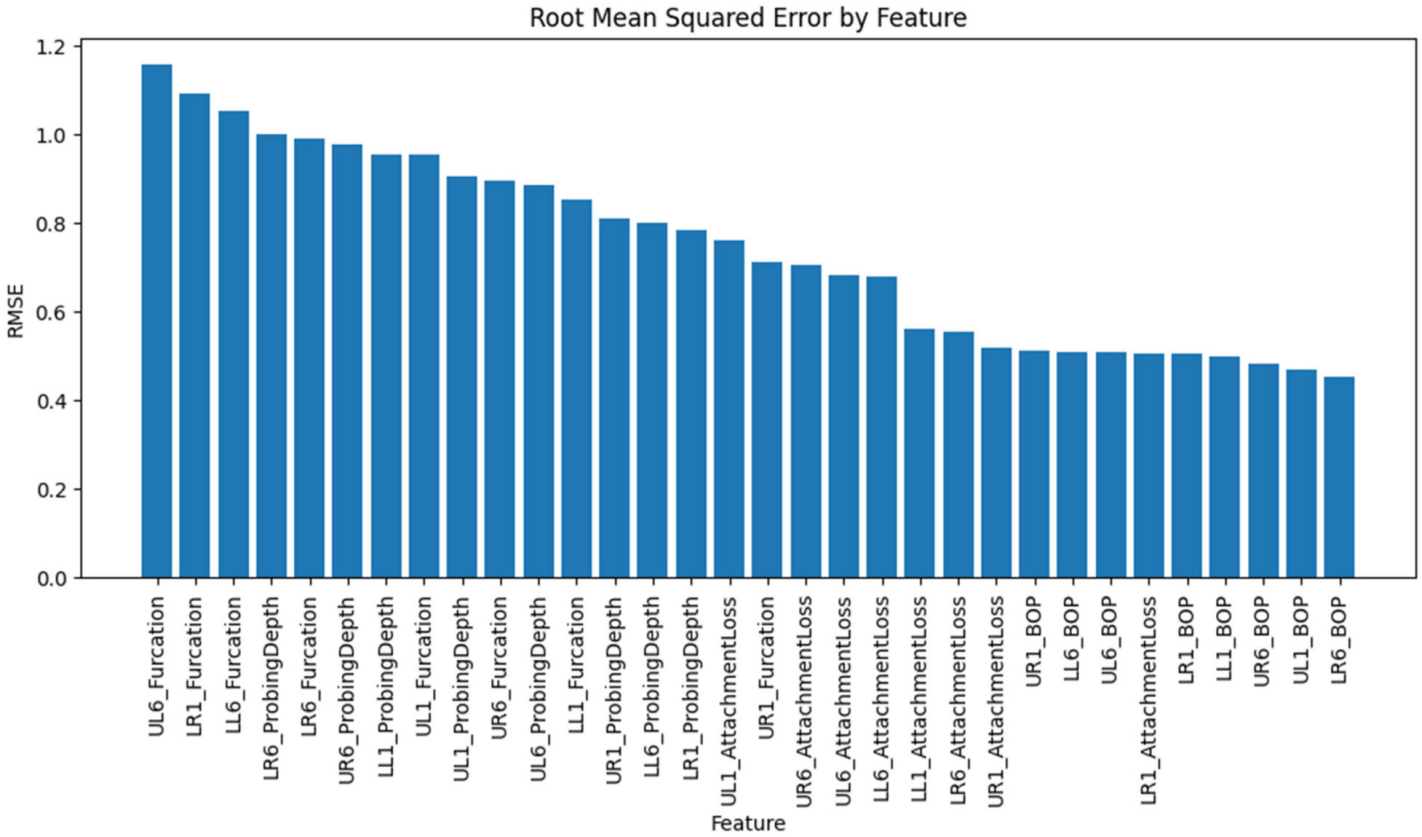
The root mean square error by feature.

**Figure 6:**
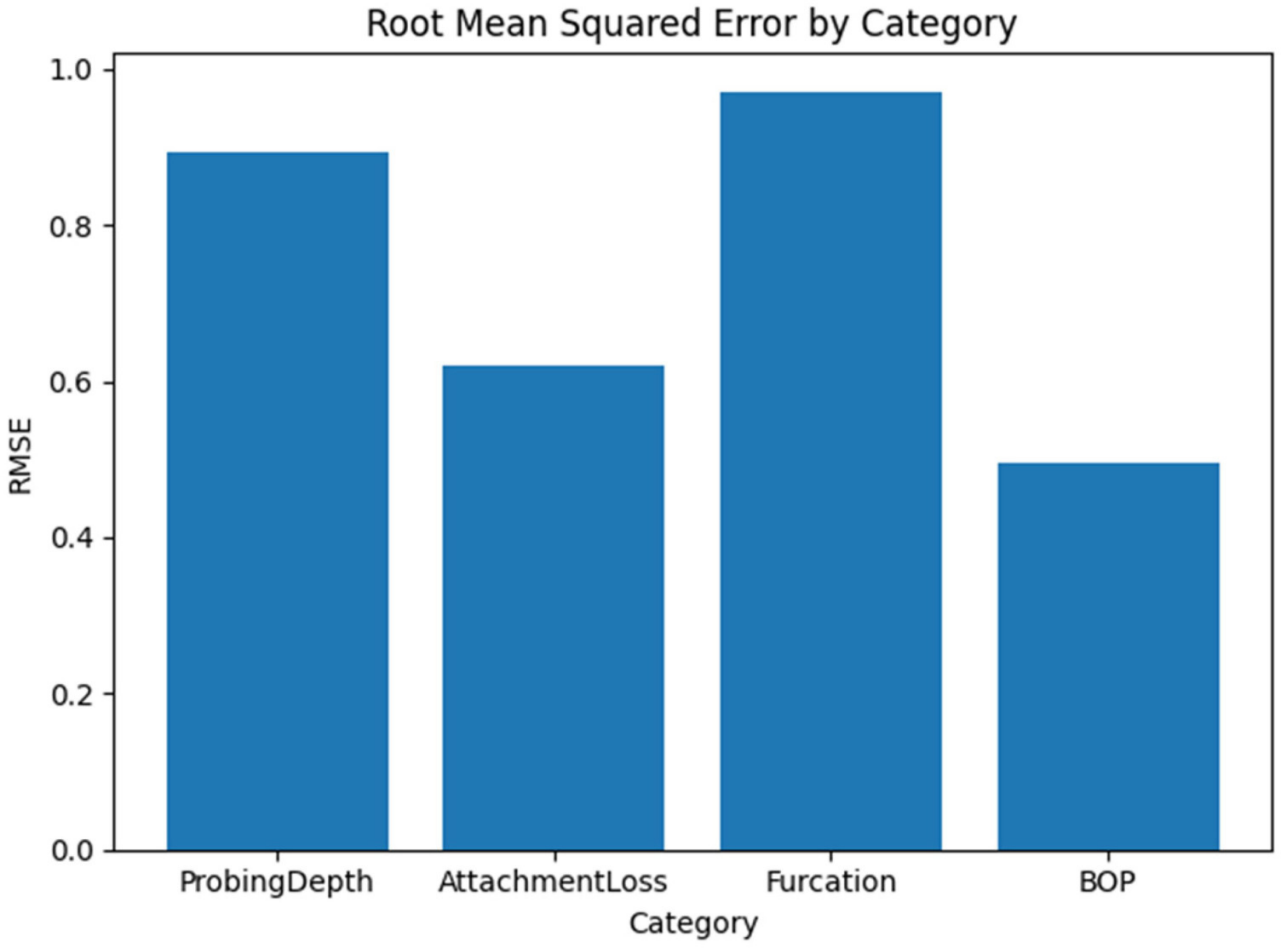
Shows the root mean square error for each category.

**Figure 7:**
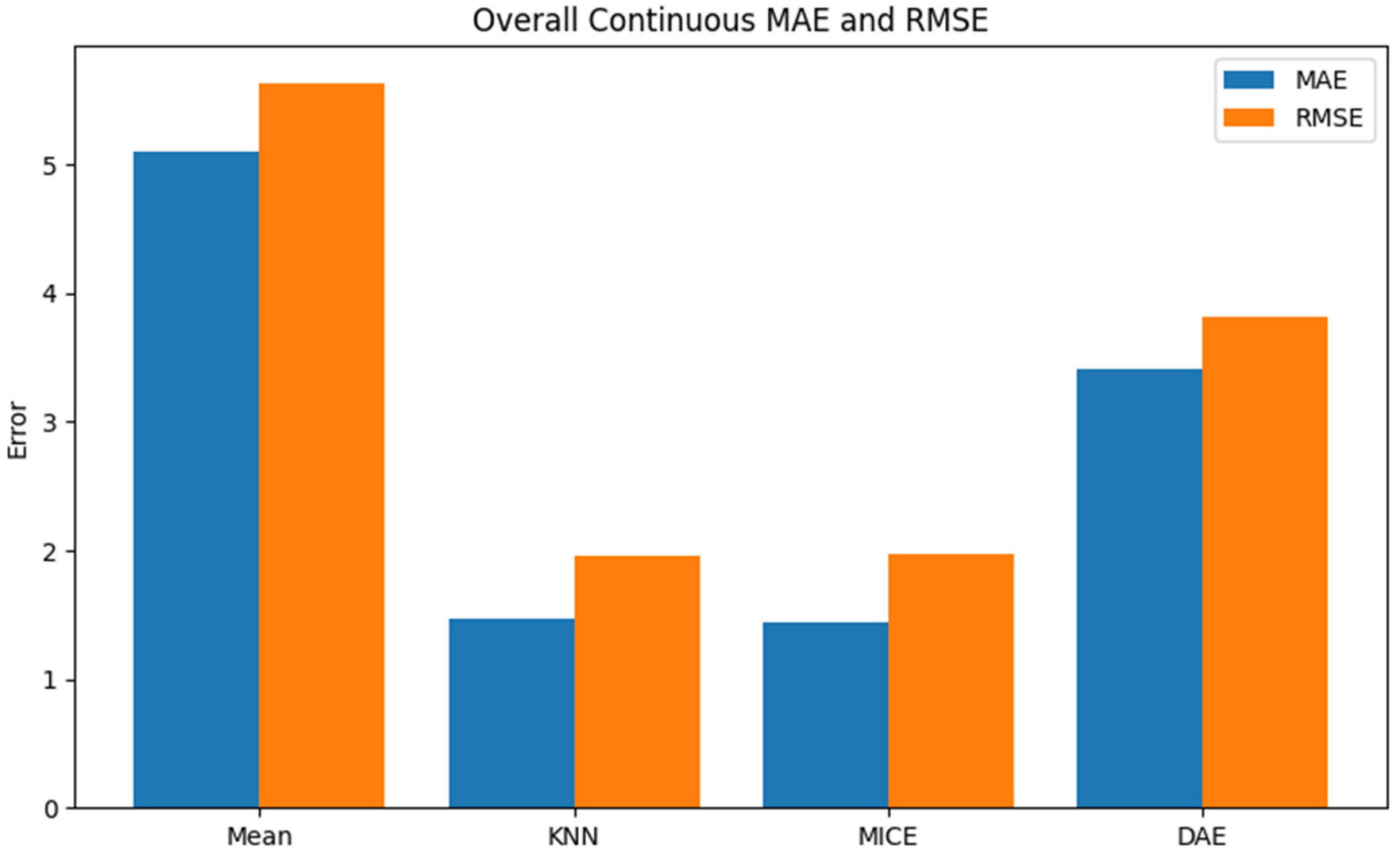
The imputation methods used for missing values.

Identifying clinical missing values is crucial in data analysis because gaps can undermine the validity of conclusions. Missing data weakens statistical power, introduces bias, and reduces representativeness. Recognizing them helps determine their mechanism—at random, at random, or not at random—and guides strategies like imputation or sensitivity analysis. Addressing missing data ensures valid results and prevents invalid conclusions. The results show that a simple denoising autoencoder effectively reconstructs missing periodontal data in a synthetic dataset, with an MAE of 0.61 and an RMSE of 0.74, indicating accurate imputation. It captures the mean and variance of continuous variables, such as probing depth and attachment loss, and correctly estimates binary variables, such as bleeding on probing. However, ordinal variables, such as furcation involvement, pose challenges because they are inherently discrete. Distribution comparisons reveal that reconstructed values are plausible and these findings suggest that the model captured the underlying structure in the synthetic dataset, but we did not formally evaluate stability using repeated splits or external validation. Thus, results indicate effective reconstruction under controlled conditions, not proof of representation generalization. Stable training loss indicates successful minimisation of reconstruction error and avoidance of overfitting. Evaluation metrics show that generative imputation produces accurate estimates of missing clinical data. On the synthetic dataset, the denoising autoencoder accurately reconstructed probing depths, attachment loss, furcation involvement, and bleeding with low error (MAE ≈ 0.61, RMSE ≈ 0.74). However, on the real 200-patient dataset, it performed poorly, while MICE and KNN had much lower errors (MAE ≈ 1.4–1.5 vs. ≈ 3.4 for the autoencoder) and nearly perfect agreement for disease severity. The real data, similar to other studies involving 200 untreated periodontitis cases, exhibited heterogeneous distributions and structured missingness, which posed a challenge to the autoencoder. These differences indicate that the synthetic cohort didn't capture all real-world data complexities, suggesting that multi-head generative models require ordinal/binary loss functions and patient-covariate conditioning. Future work should explore deeper or diffusion-based models on larger, diverse datasets to improve real-world performance. Finally, the ethical implications of reconstructing missing patient data should be considered (Figures 2–7) (Table 1).

One of the primary strengths of the generative approach is its ability to capture complex, non-linear relationships among variables. Unlike simple imputation methods that operate on a variable-by-variable basis (e.g., mean imputation or univariate regression), the autoencoder learns from the joint distribution of all variables. This enables it to infer missing values based on multiple correlated features, resulting in more realistic imputations. Additionally, the generative framework can be extended to incorporate uncertainty in the reconstructed values, for example, by using a variational autoencoder that models the latent space as a probability distribution. Such models can provide confidence intervals for imputed values and support downstream analyses that account for the uncertainty introduced by imputation. A previous study demonstrated that the Tracking-Removed Autoencoder (TRAE) enhances missing-value imputation by dynamically restructuring hidden-neuron inputs to reduce self-copying and improve cross-attribute learning. Its training scheme treats missing values as variables, allowing them to participate in model optimization, leading to superior performance on UCI datasets with complex missing patterns.

The flexibility of the generative architecture also allows it to handle high-dimensional data. Periodontal datasets often include hundreds of tooth-site variables, and traditional imputation techniques may struggle with issues such as multicollinearity or high dimensionality. In contrast, autoencoders compress the data into lower-dimensional latent representations, reducing computational complexity and capturing essential structure. This property is particularly beneficial when integrating periodontal data with other modalities such as radiographs, microbiome profiles, or genetic information. Cross-modal generative models can learn relationships between diverse data types, facilitating holistic analyses (9–11).

Another advantage is the model's generalisability to new datasets. Once trained on a representative dataset, the autoencoder can be applied to other datasets with similar variable structures. Transfer learning or fine-tuning can adapt the model to different populations or clinical settings. This reduces the need for repeated manual imputation and ensures consistent handling of missing data across studies. In large epidemiological cohorts, where manual data cleaning is infeasible, generative imputation provides an automated solution that scales to millions of records similar to one previous study assessed the accuracy and efficiency of imputation methods, including missForest, GAIN, and MICE, for handling missing data in two clinical datasets on diabetes and hypertension, with missing data rates of 20% and 50%. GAIN outperformed MICE and matched missForest at 20%, but was more accurate at 50%. It was especially effective for skewed continuous and imbalanced categorical variables, and faster than missForest. Results indicate that GAIN is promising for imputing missing data in large clinical datasets due to its accuracy, resilience to high levels of missingness, and speed (3, 5).

Despite its promise, the generative imputation approach has limitations. The synthetic dataset used captures plausible distributions but may not accurately reflect the complexity of real-world periodontal data, which can exhibit non-random missingness, variable clusters, or unobserved factors. Model performance may vary across different missingness mechanisms; thus, future work should evaluate the model on diverse anonymized clinical datasets. Our simple model, with one hidden layer and continuous outputs, is effective for demonstration but could be improved with more sophisticated architectures. Variational autoencoders, conditional variants, adversarial autoencoders, GANs, and diffusion models offer potential for better imputation, especially for discrete variables, though adaptation is needed. When training, filling missing entries with column means may introduce bias; alternatives include masking and modifying the loss function. Hyperparameter tuning and regularisation could enhance performance beyond the fixed settings used here. Evaluation metrics like MAE and RMSE measure average errors but do not capture the distribution or clinical significance of errors. Future assessments should consider clinical thresholds, binary variable metrics, comparisons with clinicians, and robust validation methods (6).

Reconstructing missing periodontal records with generative models has practical benefits. It allows larger sample sizes, improves statistical power, and reduces bias in retrospective studies. Complete datasets enhance machine learning accuracy and generalisability, as missing data hampers predictive performance. Imputation also aids meta-analyses and study integration by harmonizing variables and minimizing heterogeneity caused by missing data. Generative imputation supports clinical decisions by completing patient profiles, crucial for periodontal treatment planning. Missing data can lead to incomplete diagnoses. Automated methods fill gaps, enabling clinicians to reconstruct charts and identify disease patterns. Quantifying uncertainty in these values alerts clinicians to potentially unreliable reconstructions, prompting further exams or cautious interpretation. Third, the workflow developed in this study can be integrated into electronic health record systems to handle missing entries automatically. As dental practices adopt digital charting, implementing generative imputation modules can enhance data quality in real-time. Such integration would require careful validation and monitoring to ensure that imputed values do not mislead clinicians or misrepresent patient conditions. Transparency about which values are imputed and which are measured is essential for ethical and informed use of reconstructed data in clinical care. Several avenues for future research can build upon this work. One direction is to apply more advanced generative models, such as conditional variational autoencoders or diffusion models, to periodontal and other dental datasets. Incorporating prior knowledge about the ordinal nature of certain variables could improve the handling of furcation involvement and similar measurements. Experimenting with different noise corruption schemes and model architectures may yield better reconstructions (8, 12–15).

Evaluating the performance of generative imputation models on real-world clinical datasets is another important step. Such an evaluation should account for different missingness mechanisms (MCAR, MAR, MNAR) and the potential influence of unobserved variables. Collaborations with dental clinics and research centres could provide de-identified datasets for testing. Comparing generative imputation with traditional methods (e.g., multiple imputation, k-nearest neighbour imputation, matrix factorisation) in terms of accuracy, computational efficiency, and impact on downstream analyses would provide valuable insights (16–18). The lack of explicit demographic–clinical dependency modelling may have reduced data heterogeneity, simplifying reconstruction. This structure could explain the autoencoder's better performance on synthetic than real data. Future research should use biologically informed sampling to mimic epidemiological relationships better and improve synthetic realism. A key limitation is the lack of hyperparameter optimization or sensitivity analysis. Network depth, regularization, dropout, and training schedules weren't explored. The modest sample size and architecture may be suboptimal. Future work should include hyperparameter tuning, cross-validation, and architecture search to assess robustness and generalizability.

This study highlights a divergence between synthetic and real-world performance. The denoising autoencoder achieved high accuracy on synthetic data but was outperformed by approaches such as MICE on real clinical data. This difference likely stems from data manifold complexity: synthetic data was generated under simplified assumptions, reducing heterogeneity, while real datasets exhibit correlated dependencies, structured missingness, measurement variability, and mixed distributions that challenge simple model architectures. The autoencoder assumes smooth, continuous reconstructions, which may poorly model discrete variables like furcation involvement. Limited sample size and lack of hyperparameter tuning likely constrained generalization. These factors contribute to the gap between synthetic feasibility and real-world robustness. Synthetic data evaluation benefits from aligned data-generating assumptions; its known, simple distribution may have artificially eased reconstruction. Real-world testing provides a tougher benchmark, and the better performance of statistical methods under missing data underscores the need for context-specific evaluation.

While imputation can enhance data completeness and analysis, it introduces values that were not directly measured. Researchers and clinicians must ensure that imputed values are clearly flagged and that interpretations consider the uncertainty in these reconstructed data. Regulatory frameworks for healthcare data may need to address the use of synthetic or imputed values in clinical decision-making, research publications, and patient communication.

## Conclusion

This study presented a workflow for reconstructing missing periodontal records using generative modelling. We created a synthetic dataset, induced missingness, and trained a denoising autoencoder to impute missing data. The workflow was summarized in a diagram. Evaluation with mean absolute error and root mean squared error showed accurate, plausible imputations across variables. Category analysis revealed that furcation involved the most missingness; bleeding on probing, the least. Feature analysis identified challenging variables for reconstruction. The approach offers a promising solution for managing missing data, capturing complex relationships, generalizing to new datasets, and extending to uncertainty and multimodal data. Although our method employed a simple architecture and synthetic data, it can be applied to more advanced models and real-world data. Future work should explore advanced models, diverse clinical contexts, and integration with electronic health records, while also considering ethical implications and the transparency of imputed data.

## Data Availability

The raw data supporting the conclusions of this article will be made available by the authors, without undue reservation.
